# Hyperpolarized [1-13C]lactate flux increased in the hippocampal region in diabetic mice

**DOI:** 10.1186/s13041-019-0505-9

**Published:** 2019-11-01

**Authors:** Young-Suk Choi, Jae Eun Song, Jong Eun Lee, Eosu Kim, Chul Hoon Kim, Dong-Hyun Kim, Ho-Taek Song

**Affiliations:** 1 Department of Radiology and Research Institute of Radiological Science, Yonsei University College of Medicine, Seoul, 03722 Republic of Korea; 2 Department of Electrical and Electronic Engineering, Yonsei University, Seoul, 03722 Republic of Korea; 3 Department of Anatomy, Yonsei University College of Medicine, Seoul, 03722 Republic of Korea; 4 BK21 PLUS Project for Medical Sciences, Yonsei University College of Medicine, Seoul, 03722 Republic of Korea; 5 Department of Psychiatry, Institute of Behavioral Science in Medicine, Yonsei University College of Medicine, Seoul, 03722 Republic of Korea; 6 Department of Pharmacology, Yonsei University College of Medicine, Seoul, 03722 Republic of Korea

**Keywords:** Brain metabolism, Hyperpolarized 13C, Pyruvate metabolism, Diabetes, ATP citrate lyase, Magnetic resonance spectroscopy

## Abstract

Increasing evidence suggests there is a relationship between cognitive impairment and metabolic dysfunction. Diabetes is a chronic disease, and metabolic factors affecting brain metabolisms, such as serum glucose, insulin, and glucagon, are altered according to disease progression. In our previous study, we applied hyperpolarized [1-^13^C] pyruvate magnetic resonance spectroscopy in prediabetic mice after feeding them a 60% high-fat diet (HFD) for 6 months. Ultimately, we detected significantly increased [1-^13^C]lactate conversion in the whole brain and an almost five-fold increased [1-^13^C]lactate/pyruvate ratio in the hippocampal region. In the present study, we induced diabetes in mice by injecting streptozotocin and feeding them an HFD for 6 months. Unlike in prediabetic mice, [1-^13^C]lactate conversion in the diabetic mice did not differ from that in the control group, but [1-^13^C]lactate/total ^13^C ratio showed an almost 1.4-fold increase in the hippocampal region. We measured the amount of the lactate and mRNA levels of glucose transporters from isolated hippocampus and cortex samples. In the hippocampus, significantly decreased GLUT1 mRNA levels and increased lactate were detected, suggesting an inconsistency between glucose and pyruvate metabolism. Pyruvate can be produced from oxaloacetate as well as glucose. We investigated ATP citrate lyase (ACLY) because it cleaves citrate into oxaloacetate and acetyl CoA. Phosphorylated ACLY (Ser455), the active form, was increased in both hippocampus and cortex samples of mice injected with streptozotocin and fed an HFD. Also, phosphorylated ACLY/total ACLY showed a positive correlation with lactate amount in the hippocampus. Our results suggest that the brain has different responses to diabetic progression, but, in the hippocampus, maintains metabolic alteration toward increasing lactate production from the prediabetic to the diabetic stage. We suggest that ACLY-mediated pyruvate be used to support lactate levels in the hippocampus in cases of limited glucose availability.

## Introduction

Diabetes has been recognized as a risk factor for Alzheimer’s disease. According to epidemiological research, patients with diabetes demonstrate dementia at a rate that is two to three times higher than that of those without diabetes [1, 2]. Also, neuroimaging has revealed the appearance of cortical atrophy and a decrease in hippocampal volume in type 2 diabetes that is similar to that seen in the brains of Alzheimer’s disease patients [3, 4]. Furthermore, results obtained in animal studies show that diabetic animal models progressed to cognitive decline regardless of the method to induce diabetes, such as genetic factors (d*b*/d*b* mice), beta cell damage by streptozotocin, or feeding with a high-fat diet (HFD) [5–7]. Even though the literature suggests a clear relationship between diabetes and Alzheimer’s disease, how exactly diabetes induces cognitive decline is not yet clearly understood. As insulin resistance is the major hallmark of type 2 diabetes, impaired insulin action in the brain might engender cognitive impairment. Actually, the cerebrospinal fluid/serum insulin ratio was reported to be reduced in both type 2 diabetes and Alzheimer’s disease [8, 9], and intranasal-delivered insulin improved cognitive function in a patient with diabetes without changing serum glucose level [10, 11], supporting the suggestion that insulin resistance induced cognitive decline. However, there are conflicting results that mice having impaired insulin receptors developed insulin resistance but did not experience accelerated cognitive decline in an Alzheimer’s disease model [12]. In addition, elevated serum glucose levels in people without diabetes were associated with the development of dementia [13], while acute hyperglycemia increased amyloid beta levels in the hippocampal interstitial fluid in an Alzheimer’s disease model [14], leading to the implication that hyperglycemia could induce cognitive impairment as well. Given the fact that diabetes is a chronic disease and metabolic factors such as serum glucose, insulin, and glucagon are altered by disease progression [15, 16], new information about how the brain responds to disease progression could provide insight into the development of Alzheimer’s disease in patients with diabetes.

Hyperpolarized ^13^C magnetic resonance spectroscopy (MRS) can detect in vivo metabolism with 10,000-fold increased sensitivity and can trace metabolic fate by injecting ^13^C metabolic substrate [17, 18]. Pyruvate is the important metabolic product of glycolysis and is located between cytoplasmic metabolism by converting lactate and mitochondrial metabolism by converting acetyl CoA [19]. In a previous study, we reported metabolic changes achieved using hyperpolarized [1-^13^C] pyruvate MRS in the brain of prediabetic mice by feeding them an HFD for 6 months. These mice experienced weight gain with hyperglycemia, but there was no difference in glucose tolerance test outcomes. They showed significantly increased hyperpolarized lactate conversion in the whole brain, and the medial temporal lobe containing the hippocampus was the prominent region for this metabolic alteration [20], suggesting that increased lactate production is associated with cognitive decline. In this study, we investigated whether the metabolic alterations seen in prediabetes are maintained or changed according to disease progression. To induce beta cell damage, mice were injected with 100 mg/kg of streptozotocin and fed a 60% HFD for 6 months, then subjected to a hyperpolarized [1-^13^C] pyruvate MRS study in the brain.

## Materials and methods

### Animal procedures

Seven weeks old male ICR mice were purchased from Japan SLC, a branch of Charles River Laboratories (Shizuoka, Japan). The mice were fed either a normal diet (ND; 5053, 13.1 kcal % fat; PicoLab, Tokyo, Japan) or an HFD (fat 54.3% kcal of lard, 5.6% kcal of soybean oil; D12492; Research Diets Inc., New Brunswick, NJ, USA) for 24 weeks. All animal procedures were carried out according to a protocol approved by the International Animal Care and Use Committee (IACUC) of the Yonsei University Animal Research Center (YLARC; permission no. 2017–0018) following the National Institutes of Health guidelines. All animals were maintained in a specific pathogen-free facility of the YLARC with a well-controlled temperature (23 °C) and light cycle (12 h of light and 12 h of dark) and easy access to water and food.

To induce type 2 diabetes, mice that were fed an HFD (60% kcal fat) for 1 month received a single injection of a low dose of streptozotocin (100 mg/kg/ip) dissolved in citrate buffer (pH.4.4) and then were maintained continuously on an HFD diet [21].

### Intraperitoneal glucose tolerance test (IPGTT)

The intraperitoneal glucose tolerance test was performed at 12 weeks after a high-fat diet. Mice were fasted overnight and injected with glucose (1 g/kg/ip, dissolved in saline) in the morning. Blood sampels were taken from the tail vein and blood glucose levels were measured using a glucometer at 0, 30, 60, and 120 min after the bolus [10].

### Hyperpolarized ^13^C MR spectroscopy

Mice were fasted for 4–5 h before hyperpolarized ^13^C MR spectroscopy. A total of 26.7 mg of [1-^13^C] pyruvic acid (Cambridge Isotope, Tewksbury, MA, USA) was mixed with 15 mM of trityl radical OX-063 (Oxford Instruments, Oxford, UK) and 0.75 mM of gadoterate meglumine (Dotarem®; Guerbet, Villepinte, France). This [1-^13^C] pyruvic acid sample was hyperpolarized using a dynamic nuclear polarization system (HyperSense®; Oxford Instruments, Oxford, UK) and dissolved with 3.8 mL of Tris/EDTA-NaOH buffer, resulting in 79 mM of pyruvate (pH: 7.5) with a polarized level of approximately 20%. We drew 350 μL of hyperpolarized [1-^13^C] pyruvate into a syringe for in vivo MRS. In-vivo experiments were performed on a 9.4 T Bruker BioSpec 94/20 small animal imaging MRI scanner (Bruker Biospin MRI GmbH, Ettlingen, Germany) equipped with a ^13^C receive-only mouse brain surface coil. T2 weighted ^1^H images were acquired for 7 coronal slices with a thickness of 3.5 mm. Afterwards, hyperpolarized 13C free induction decay (FID) chemical shift image was obtained using centric-ordered phase encoding with a flip angle of 10°, field of view of 18 × 24 mm^2^, matrix size of 18 × 24, slice thickness of 3.5 mm, spectral bandwidth of 6510.4 Hz, spectral points of 512, and spectral resolution of 12.7 Hz. Imaging acquisition started 18 s after the start of the intravenous injection of pyruvate, and the total acquisition time was 35 s. Each FID signal was zero-filled with three times of spectral points and filtered with exponential apodization function. In voxel-based analysis, we calculated the hyperpolarized [1-^13^C]lactate/total ^13^C ratio to compensate for the inhomogeneous substrate perfusion and coil sensitivity [22]. All data were processed using MATLAB-based analysis (R2017a; MathWorks, Natick, MA, USA).

### Tissue sample preparation

After finishing hyperpolarized ^13^C MR spectroscopy, the mice were maintained under the same experimental condition for almost 3–6 days and then sacrificed via CO_2_ inhalation after fasting for nearly 4 h. Brains were collected and placed in saline, and the hippocampus and cortex were carefully isolated. Tissues were frozen in liquid nitrogen and stored at − 80 °C. For in vitro study, tissues were homogenized in phosphate-buffered saline (PBS) without calcium and magnesium and divided into three vials for RT-PCR, western blot, and lactate measurement, respectively.

### Assessment of lactate level

Lactate levels were measured using the lactate colorimetric assay kit (Biovision, San Francisco, CA, USA). Tissue samples were lysed using an NP-40 buffer. After measuring BCA-based protein concentrations, 40 μg of lysate was used according to the manufacturer’s instructions to detect lactate concentration. The absorbance was measured at 450 nm.

### Western blot

Protein was isolated using ice-chilled RIPA lysis and extract buffer (Thermo Fisher Scientific, Waltham, MA, USA) containing phosphatase inhibitors (5 mM of β-glycerophosphoric acid, 10 mM of NaF, and 1 mM of Na3VO4), 1 mM of PMSF, and protease inhibitor cocktail (Sigma Aldrich, St. Louis, MO, USA). Homogenate containing 15 μg of protein was subjected to 10% SDS-PAGE under reducing conditions. The proteins were transferred to PVDF membranes in transfer buffer and then separated at 400 mA for 2 hours at 4 °C. The Western blots were subsequently incubated for 2 hours with 5% skim milk at room temperature and then incubated overnight with a 1:1000 dilution of anti-LDHA (NBP1–48336; Novus Biologicals, Centennial, CO, USA), anti-LDHB (AB85319; Abcam, Cambridge, UK), phospho–ATP–citrate lyase (Ser455) (4331; Cell Signaling Technology, Danvers, MA, USA), ATP-Citrate lyase (4332, Cell Signaling Technology, MA, USA) anti-β-actin (sc-47,778; Santa Cruz Biotechnology, Dallas, TX, USA), Then, the blots were washed twice with Tween 20/Tris-buffered saline (TTBS) and incubated with a 1:3000 dilution of horseradish peroxidase-conjugated secondary antibody for 2 hours at room temperature. After washing three times with TTBS, blots were developed using WEST-SAVE Up luminol-based ECL reagent (ABfrontier, Seoul, Korea). The membranes were analyzed using the ImageJ software (National Institutes of Health, Bethesda, MD, USA).

### RT-PCR

Trizol reagent (Gibco Laboratories, Gaithersburg, MD, USA) was used to extract total RNA by following the manufacturer’s instructions. TOYOBO ReverTra Ace® qPCR RT Master Mix (Toyobo, Osaka, Japan) was used to obtain complementary DNA using 800 ng of total RNA. PCR reaction was performed using ready-2x-Go Taq (NanoHelex, Daejeon, Korea) in the C1000 Touch™ Thermal Cycler (Bio-Rad Laboratories, Hercules, CA, USA), following the manufacturer’s guidelines. PCR products were run on a 1.5% agarose gel in TAE buffer and visualized using the MiniBIS Bioimaging system (Neve Tamin, Israel). Gene expression levels were analyzed using the ImageJ software. The primers used were as seen in Table 1.

**Table 1:** PCR primers

Gene			Cycle	size
Glut1	Forward	GGGTCTTAAGTGCGTCAGGG	30	312
NM_011400.3	Reverse	AGAGAGACCAAAGCGTGGTG		
Glut2	Forward	ACCGGGATGATTGGCATGTT	35	288
NM_031197.2	Reverse	GAACACGTAAGGCCCAAGGA		
Glut3	Forward	TGTGGTAAAAAGCAGGAGGGG	35	251
NM_011401.4	Reverse	TGTTCCTCGGGTCCTACAGA		
Glut4	Forward	TCACTAGATCCCGGAGAGCC	35	235
NM_001359114.1	Reverse	GGGTTCCCCATCCTTACGTC		
GAPDH	Forward	CCCTTAAGAGGGATGCTGCC	30	124
NM_001289726.1	Reverse	TACGGCCAAATCCGTTCACA		
β-actin	Forward	GATTACTGCTCTGGCTCCTAG	30	147
NM_007393.5	Reverse	ACTCATCGTACTCCTGCTTG		

### Statistical analysis

Statistical analyses were performed to compare the two groups: mice on a normal diet vs. streptozotocin-injected mice on an HFD using the Student’s t-test. Pearson’s correlation coefficient is used to determine the linear relationship between pACLY/ACLY ratio and lactate level in the brain tissues. All results were expressed as means ± standard error of the mean (SEM), and *p* < 0.05 was considered to be statistically significant. Statistical analysis was performed using statistical software (PRISM version 6.0; GraphPad Software, San Diego, CA, USA).

## Results

### Mice injected with streptozotocin and fed an HFD had increased fast serum glucose and showed glucose intolerance

**Fig. 1:**
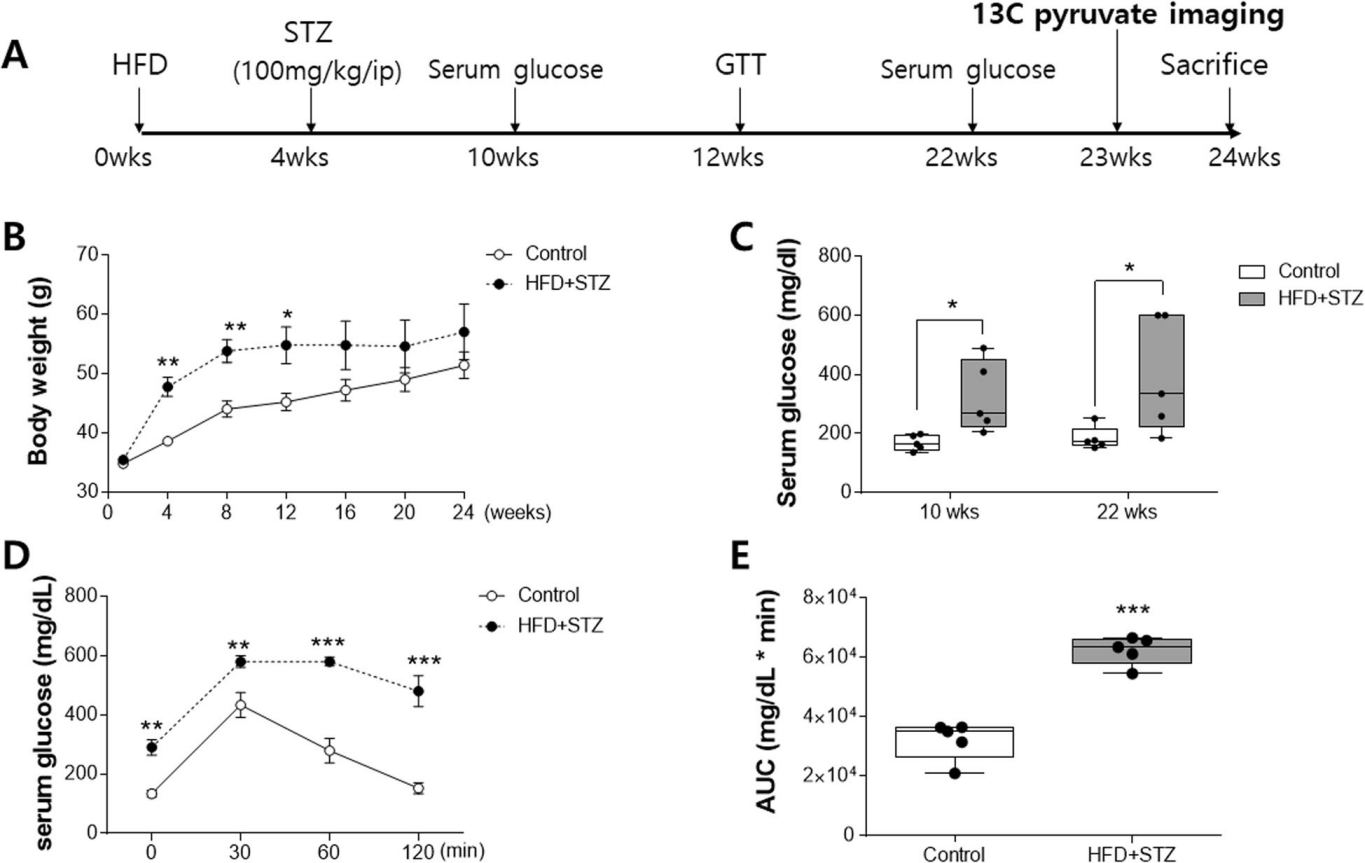
Weight gain, hyperglycemia, and impaired glucose tolerance were observed in the streptozotocin-injected mice fed an HFD (HFD + STZ). **a** Experimental schedule. **b** Body weight was measured every 4 weeks for each diet group. **c** Four-hour fasting for serum glucose level was measured at 10 weeks and 22 weeks. **d** Changes in the glucose level during the intraperitoneal glucose tolerance test (GTT). **e** The area under the curve (ACU) of the glucose level was calculated during the IPGTT. STZ; streptozotocin. IPGTT; intraperitoneal glucose tolerance test. Values are expressed as means ± SEMs (*n* = 5 for both groups). * *p* < 0.05, ** *p* < 0.01, *** *p* < 0.001

Streptozotocin-injected mice fed an HFD (HFD + STZ) showed significant weight gain (Fig. 1b) and increased fasting serum glucose levels (Fig. 1c). Also, they showed significantly impaired glucose tolerance test findings (Figs. 1d-e).

### Higher hyperpolarized [1-^13^C]lactate/total ^13^C ratio is visualized in the region containing the hippocampus in mice injected with streptozotocin and fed an HFD

**Fig. 2:**
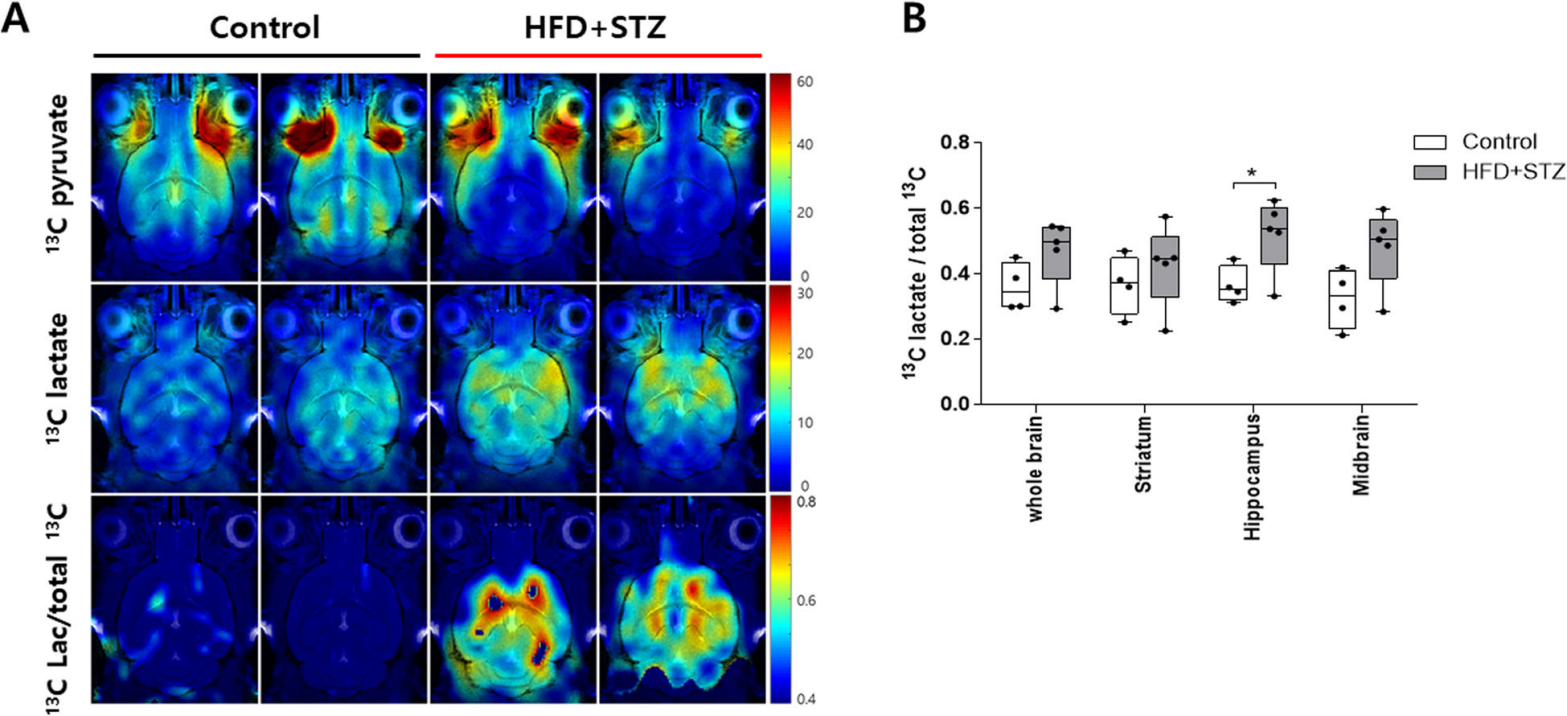
Chemical shift imaging of hyperpolarized ^13^C MRS. **a** Color maps overlaid on the ^1^H images represent [1-^13^C] pyruvate and [1-^13^C] lactate peak intensities, and [1-^13^C] lactate/total ^13^C intensity ratios. The images were acquired for 35 s from 18 s after intravenous injection of 79 mM of hyperpolarized ^13^C-pyruvate in the coronal plane with a 3.5-mm slice thickness and 1 × 1 mm^2^ in-plain resolution. White boxes indicate voxels containing hippocampus. **b** [1-^13^C] lactate/total ^13^C intensity ratios calculated each voxel of the region containing the whole brain, striatum, hippocampus, and midbrain. Values are expressed as mean ± SEMs (*n* = 4–5 for both groups). * *p* < 0.05

To investigate the brain response to the metabolic change provoked by diabetes, we performed hyperpolarized ^13^C MR chemical shift imaging in the brain. The hyperpolarized [1-^13^C] pyruvate signal revealed a downward trend, suggesting decreased cerebral perfusion. However, [1-^13^C]lactate production was still maintained and a higher [1-^13^C] lactate/total ^13^C ratio was visualized in the brains of mice injected with streptozotocin and fed an HFD (Fig. 2a). The [1-^13^C]lactate/total ^13^C ratio was significantly increased in the region containing the hippocampus in the brains of mice injected with streptozotocin and fed an HFD (Fig. 2b).

### Lactate level increased in the hippocampus of mice injected with streptozotocin and fed an HFD

**Fig. 3:**
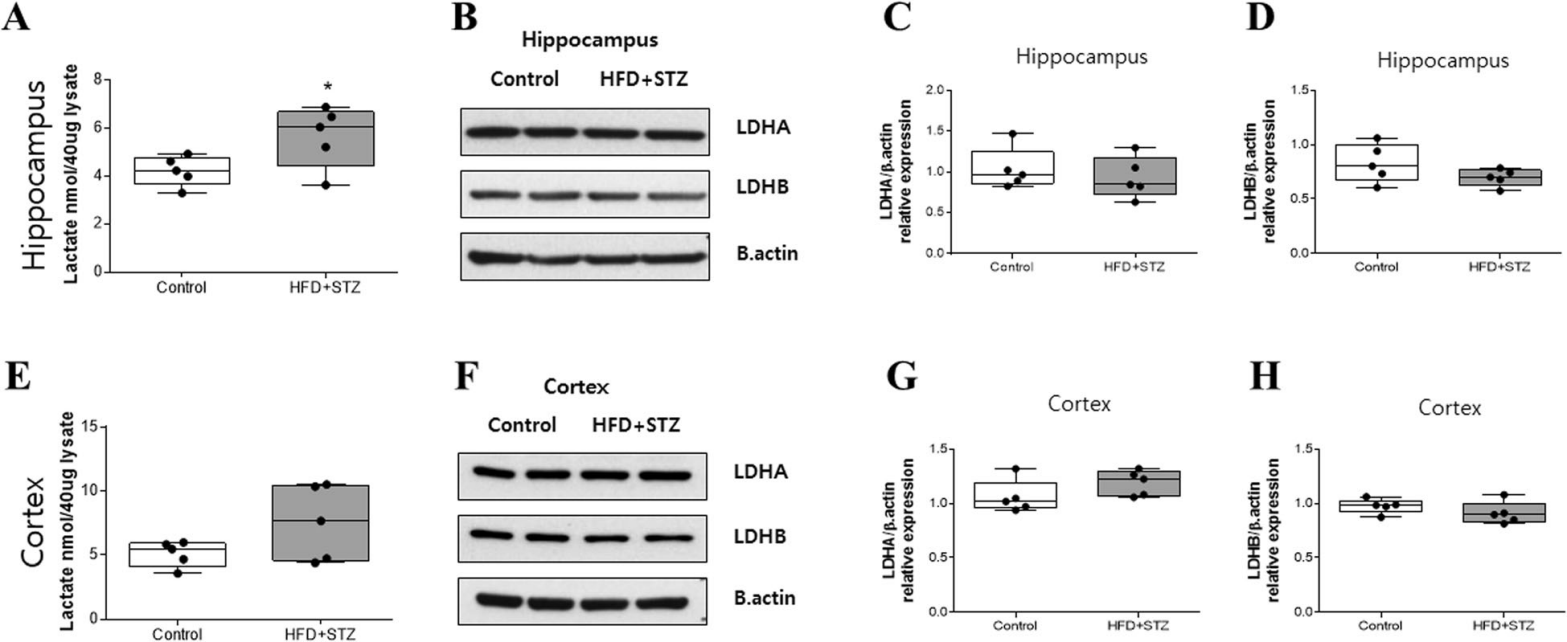
Increased lactate level detected in the hippocampus of streptozotocin-injected mice fed an HFD (HFD + STZ). **a** The amount of lactate in 40 μg of a lysate measured in the hippocampus. **b** LDHA and LDHB protein levels were measured by western blot assay. **c** LDHA and **d** LDHB protein levels were quantified by the ratio to β-actin in the hippocampus. **e** Amount of lactate in 40 μg of a lysate of the cortex. **f** Cropped images of LDHA and LDHB in the cortex. **g** Quantified LDHA and **h** LDHB in the cortex by the ratio to β-actin. Values are expressed as means ± SEMs (*n* = 5 for both groups). * *p* < 0.05

Since the signal intensity of hyperpolarized [1-^13^C] lactate reflects the amount of lactate pooled in tissue [23], we measured lactate level in the hippocampus and cortex to validate our hyperpolarized [1-^13^C] pyruvate imaging study. We found that lactate level was significantly increased in the hippocampus (Fig. 3a) but it was not significantly increased in the cortex (Fig. 3e) of mice injected with streptozotocin and fed an HFD. However, there was no difference in lactate dehydrogenase A (LDHA) or LDHB protein levels in both the hippocampus (Figs. 3b-d) and cortex (Figs. 3f-h).

### Gene expression level of glucose transporter 1 was decreased in the hippocampus of mice injected with streptozotocin and fed an HFD

**Fig. 4:**
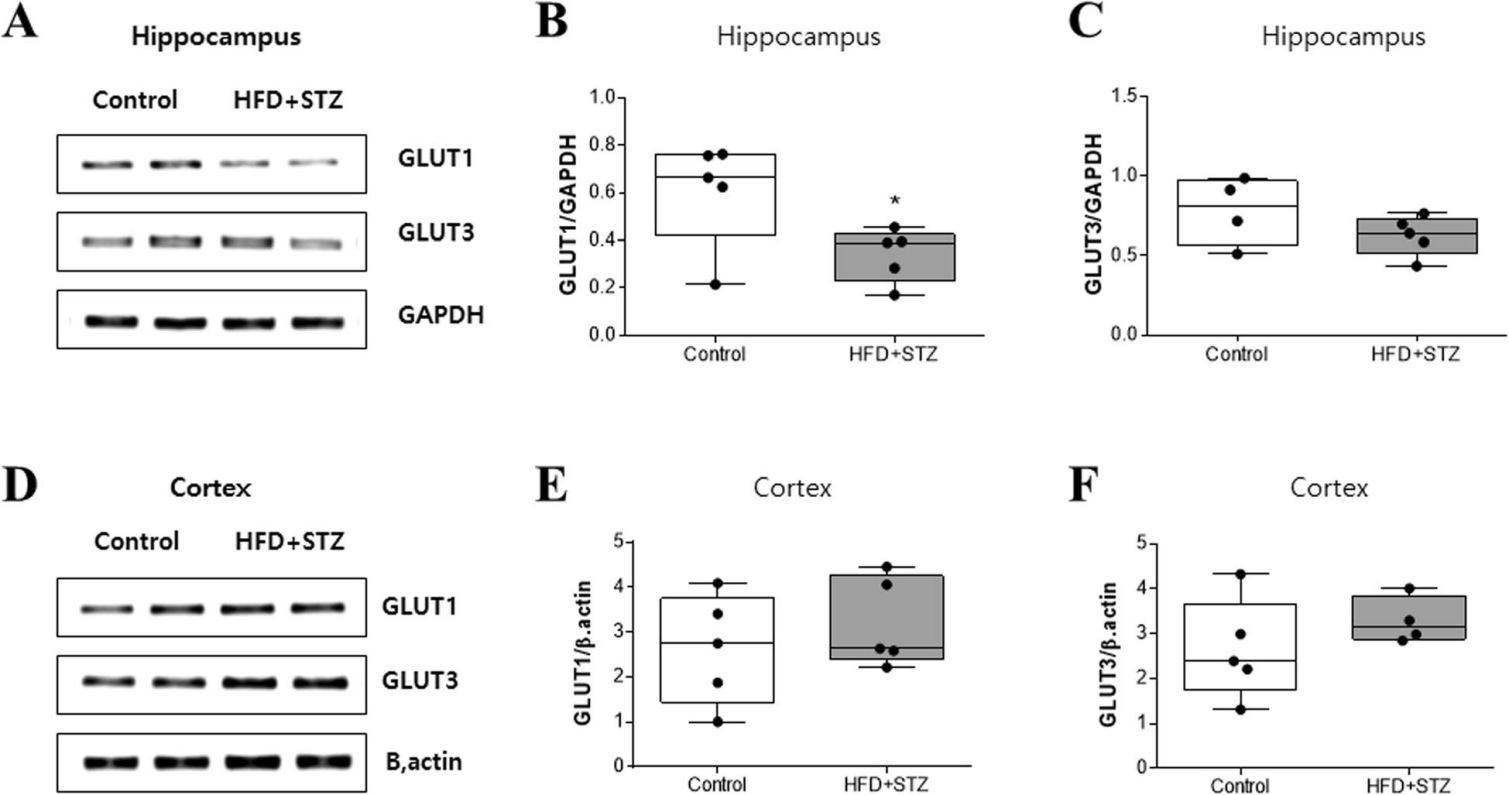
GLUT1 mRNA level was decreased in the hippocampus of the streptozotocin-injected mice fed an HFD (HFD + STZ). **a** Representative RT-PCR product bands of GLUT1, GLUT3, and GAPDH mRNA expression in the hippocampus. **b** GLUT1 and **c** GLUT3 gene expression levels were quantified by the ratio to GAPDH in the hippocampus. **d** Representative RT-PCR product bands of GLUT1, GLUT3, and GAPDH mRNA expression in the cortex. **e** Quantified GLUT1 and **f** GLUT3 in the cortex by the ratio to β-actin. Values are expressed as means ± SEMs (*n* = 5 for both groups). * *p* < 0.05

Since lactate is one of the major products of glucose metabolism created via glycolysis in the cytoplasm, we investigated the expression level of glucose transporters (GLUT) in both the hippocampus and cortex to elucidate whether increased lactate production was the result of increased glucose uptake in the mice injected with streptozotocin and fed an HFD. GLUT1 expression was decreased in the hippocampus of the mice fed an HFD (Fig. 4a and b) but no difference was seen in the cortex (Fig. 4d and e). On the other hand, the GLUT3 expression level was similar in both groups (Fig. 4a, c, d, and f). Separately, GLUT2 and 4 were hardly detected in both the hippocampus and cortex (data not shown).

### Phosphorylated ATP citrate lyase (ACLY) increased in mice injected streptozotocin and fed an HFD

**Fig. 5:**
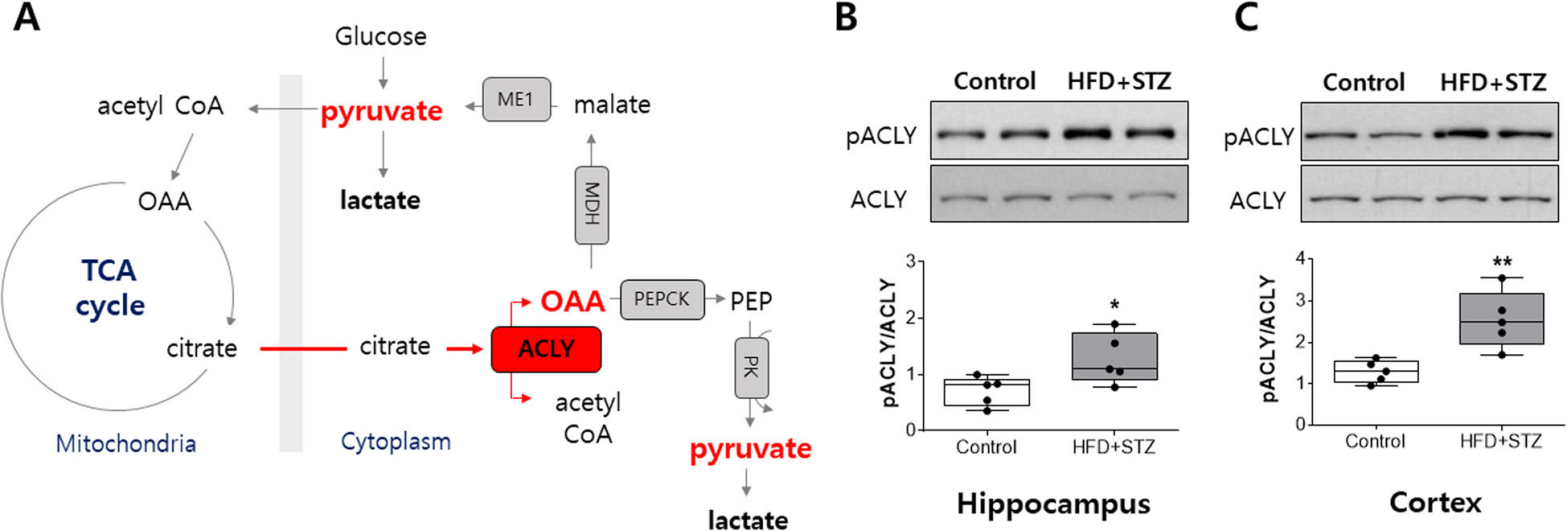
Phosphorylated ACLY (Ser455) increased in both the hippocampus and cortex in the streptozotocin-injected mice fed an HFD (HFD + STZ). **a** Expected metabolic pathway to supply pyruvate via ACLY. OAA; oxaloacetate, ME1; malic enzyme1, MDH; malate dehydrogenase, PEPCK; phosphoenolpyruvate carboxykinase, PK; pyruvate kinase, ACLY; ATP citrate lyase. **b** Phosphorylated ACLY (Ser455) and total ACLY protein levels were measured by western blot assay. Phosphorylated ACLY (Ser455) was quantified by the ratio of total ACLY; representative cropped images in the hippocampus and **c** in the cortex are presented. Values are expressed as means ± SEMs (*n* = 5 for both groups). * *p* < 0.05., ** *p* < 0.01

Even though there are conflicting results regarding glucose transporter expression level in the brain affected by diabetes [24–26], the ability to import glucose into the brain has been suggested to be decreased in a manner similar to our results shown in Fig. 4a and b. Therefore, we investigated the possible metabolic pathway to support pyruvate except for glycolysis. We focused on ATP citrate lyase (ACLY) because it converts citrate into acetyl CoA and oxaloacetate (OAA). From OAA, pyruvate can be produced via phosphoenolpyruvate carboxykinase (PEPCK) and pyruvate kinase or via malate dehydrogenase (MDH) and malic enzyme 1 (ME1) (Fig. 5a). Since the phosphorylation of ACLY on Ser455 has been reported to enhance the catalytic activity [27, 28], we measured it in both the hippocampus and cortex. Phosphorylated ACLY on Ser455 increased in both the hippocampus (Fig. 4b) and cortex (Fig. 4c), but the total ACLY level was not significantly increased.

### Lactate level correlated with phosphorylated ATP citrate lyase in the hippocampus of mice fed an HFD

**Fig. 6:**
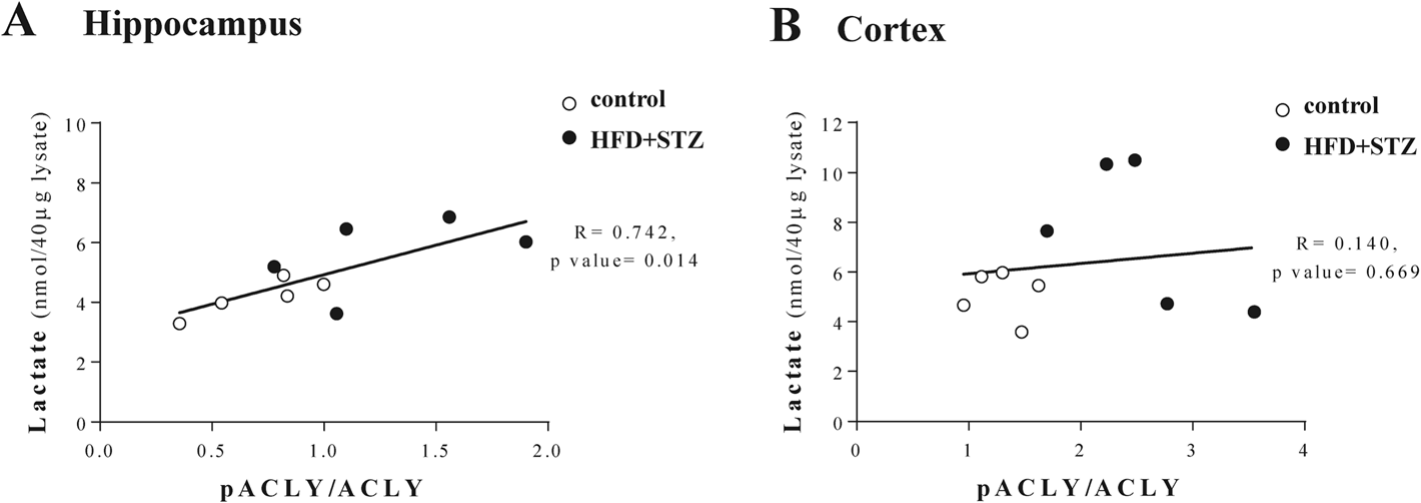
Amount of lactate showed a positive correlation with phosphorylated ACLY in the hippocampus of streptozotocin-injected mice fed an HFD (HFD + STZ). **a** Person’s correlation coefficient value calculated in the hippocampus and **(b)** in the cortex between the amount of lactate and the pACLY/ACLY ratio

To investigate the possibility that ACLY-mediated pyruvate production supports lactate production, we calculated the Pearson’s correlation coefficient value between the amounts of lactate and the phosphorylated ACLY ser455 in both the hippocampus and cortex. As shown in Fig. 6, the lactate level demonstrated a positive correlation with pACLY levels in the hippocampus but not in the cortex. This suggests that increased lactate in the hippocampus of mice injected with streptozotocin and fed an HFD may come from ACLY-mediated pyruvate production in the face of limited glucose availability.

## Discussion

In the present study, we extend our previous work by performing hyperpolarized ^13^C pyruvate in vivo imaging in diabetic mice, which have considerably impaired glucose tolerance, to compare brain response to that of control mice. Our results indicate that a significantly increased hyperpolarized lactate/total ^13^C ratio can be detected in the hippocampus, even though the GLUT1 mRNA level was decreased.

With our previous finding in the brains of prediabetic mice, the hyperpolarized ^13^C lactate flux significantly increased approximately 4.5-fold and 5-fold in the whole brain and the region containing the hippocampus, respectively [20]. However, from this study in diabetic mice, the hyperpolarized ^13^C lactate flux did not show any difference between diabetic mice and controls in the whole brain, and a significant increase of about 1.4-fold in the region containing the hippocampus was observed in diabetic mice. Therefore our results support that different brain regions respond to metabolic stress according to disease progression, and lactate production is activated in the region containing the hippocampus during prediabetic to diabetic progression, although the degree of increase is different.

Metabolic imaging studies suggest decreased ^18^F-FDG uptake occurs in Alzheimer’s disease [29], and this hypometabolism can be observed prior to the symptoms [30, 31]. Decreased FDG-PET uptake presents in both patients with prediabetes and type 2 diabetes [32, 33], suggesting that reduced glucose metabolism in the brain induces cognitive impairment. Also, impaired insulin signaling pathways [21, 34, 35] and reduced glucose transporter levels are seen in Alzheimer’s disease [36, 37] and could support that a hypoglucose metabolism leads to induce cognitive decline. However, our results suggest the inconsistency of glucose and pyruvate metabolism in the hippocampus of diabetic mice, as the level of GLUT1 expression was decreased and that of lactate was increased.

From the classical point of view, glucose is the primary energy source to be used in both neurons and astrocytes, and lactate is considered a by-product. Recently, the concept of the astrocyte–neuron lactate shuttle hypothesis has been supported with a number of research papers. This hypothesis suggests that astrocytes uptake glucose, produce lactate via glycolysis, and then release it to extracellular spaces. Meanwhile, neurons uptake lactate and use it as an energy source to make ATP using oxidative phosphorylation in the mitochondria [38]. Because astrocytes have greater metabolic plasticity than neurons, the astrocyte–neuron lactate shuttle system makes it possible for neurons to maintain their function in stress conditions such as diabetes or hypoxia [38, 39]. Therefore, the inconsistency of glucose and pyruvate metabolism in our results may reflect that astrocytes support lactate production in the case of limited glucose availability to support neuronal function. We suggest that ACLY supports lactate in the hippocampus of diabetic mice. The expression pattern of ACLY in the brain is interesting because the amount of ACLY level decreases with age, yet a relatively high expression level was detected in the hippocampus in adult mice [40], suggesting ACLY plays a role in the hippocampus. Increased levels of citrate, acetyl CoA, and lactate in the brain of streptozotocin-induced diabetic rats [41] may support the role of ACLY to produce lactate in the brain in diabetes because of ACLY cleaves citrate into acetyl CoA and OAA.

In our result, the amount of lactate showed a positive correlation with phosphorylated ACLY in the hippocampus, but not in the cortex. Several factors regulate the amount of lactate, including glycolysis, the activity of pyruvate dehydrogenase, and NAD+/NADH redox state [42, 43]. Therefore, the regional difference of those factors may result in a different correlation between phosphorylated ACLY and the amount of lactate level. In this study, we showed altered brain metabolism toward increased lactate level. However, it remains unclear as to whether the increased amount of lactate in the hippocampus induces cognitive decline or whether the altered metabolic pathway to support lactate production causes cognitive decline.

Increased ACLY activity in the brain of diabetes might be related to lipid accumulation in the brain. Fatty acid synthesis occurs in the cytoplasm by serial chain elongation of acetyl CoA. Therefore, knowing the role of ACLY in the cytoplasm and of supporting acetyl CoA is essential [27, 28]. The importance of lipid metabolism in cognitive decline is emphasized by the fact that lipid inclusion in glial cells is one of the pathologic hallmarks reported by Dr. Alzheimer [44]. Also, aging, one of the risk factors for Alzheimer’s disease, was reported to correlate with increasing lipid accumulation in both aged mice with Alzheimer’s disease and aged normal Balb/C mice [45, 46].

The other supporting evidence that lipid accumulation leads to a cognitive decline can be taken from the results obtained in the people having apolipoprotein E (ApoE) ε4 polymorphism. These individuals have a higher risk of developing Alzheimer’s disease than do those with ApoE ε3 polymorphism, the most common form. ApoE isoforms have different affinities for transporting lipid, and ApoE ε4 has been known to have a lesser degree of efficacy in comparison with APOE ε3 [47, 48]. Also, people having both diabetes and ApoE ε4 have more than twice the risk of developing Alzheimer’s disease than do those with ApoE ε4 alone [49]. Interestingly, there was no difference in cognitive function in the normal diet–fed condition between genetic mice having ApoE ε4 and ApoE ε3. However, mice having ApoE ε4 showed significantly impaired cognitive function compared to mice having ApoE ε3 when fed an HFD [50]. This finding suggests the greater importance of lipid accumulation than ApoE polymorphism in the development of Alzheimer’s disease. Therefore lipid accumulation in the brain by diabetes or metabolic stress may boost the development of cognitive decline in people having ApoE ε4. In this study, we proposed that ACLY plays a potential role in producing lactate in the diabetic state, and this metabolic adaptation may induce lipid synthesis. However, further study is required to investigate whether astrocyte promotes ACLY-mediated lactate production in the face of limited glucose supply. Also, we must further review whether ACLY activation stimulates lipid accumulation in the brain of diabetic mice.

In this work, our hyperpolarized ^13^C pyruvate imaging shows that different brain regions respond differently to metabolic stress according to diabetes progression, but significantly higher hyperpolarized ^13^C lactate flux visualized in the hippocampus of diabetes. With the inconsistency of GLUT1 mRNA expression level and amount of lactate in the hippocampus of diabetes, we suggest that ACLY supplies lactate in the case of limited glucose availability to support neuronal function. Lipid accumulation in glial cells was one of the pathologic hallmarks of Alzheimer’s disease, and the role of ACLY for fatty acid synthesis is essential. Thus, our result would provide a clue to investigate further the relationship between lipid accumulation in the brain of diabetes and Alzheimer’s disease progression.

## Data Availability

All data generated or analyzed during this study are included in this published article.
